# Analysis of prevalence of PTSD and its influencing factors among college students after the Wenchuan earthquake

**DOI:** 10.1186/1753-2000-7-1

**Published:** 2013-01-19

**Authors:** Yan Fu, Yongshun Chen, Jin Wang, Xiaohui Tang, Jieyun He, Miaorui Jiao, Chunhua Yu, Guiying You, Junying Li

**Affiliations:** 1 Division of Thoracic Oncology, West China Hospital, Cancer Center, West China School of Medicine, Sichuan University, 37, Guoxue Lane, Chengdu, 610041, China; 2 Zhengzhou University Affiliated Tumor Hospital, Henan Tumor Hospital, Zhengzhou, China; 3 Chengdu Aier Eye Hospital, Chengdu, China; 4 Hong Kong Polytechnic University, Hong Kong, China

**Keywords:** China, Disaster, Mental health, PTSD, Prevalence

## Abstract

**Background:**

This study explored the prevalence and severity of post-traumatic stress disorder (PTSD) in college students who lived in earthquake center one year after the Wenchuan earthquake on May 12, 2008, the factors affecting the prevalence of PTSD was also investigated.

**Methods:**

2987 students studying at the senior normal school in Tibetan autonomous region which was one of the most devastated regions were selected for this study. The PTSD Checklist-Civilian Version (PCL-C) was used as a screening instrument.

**Results:**

A total of 420 cases (14.1%) were diagnosed with PTSD, among which mild, moderate, severe and extreme symptoms were reported in 122, 185, 106 and 7 cases, respectively. The PTSD prevalence in college students lived in the severely affected area was significantly higher than that in the less severe area (P < 0.001). According to the multivariate logistic regression analysis, the students who were injured in the earthquake, those lost their first degree relative, and those confronted with dead bodies were more likely to express PTSD. Male students were more prone than female students to develop PTSD. However, the students who received psychological tutorship were less prone to express PTSD.

**Conclusions:**

At one year after the earthquake, the PTSD rate in college students in the severely affected area was high. The social support, psychological help and rehabilitation project should be strengthened to improve their ability to cope with the trauma.

## Background

The 8.0 earthquake on Richter scale occurred in Wenchuan county of Sichuan province on May 12, 2008 had caused shock and grief worldwide. 69227 people were killed during the earthquake and more than 374000 injuries were reported officially. The immense destruction also brought severe harms to social economic development and building safety. Survivors who experienced uncommon disasters will have stress responses such as being at loss, torpor, anxiety and depression. Most people will gradually recover after adjusting for several weeks or months. However, some people will not recover due to excessive psychological trauma, therefore repeatedly having symptoms of numbness, increased alertness, and problems of memory and cognition, which is typical of post-traumatic stress disorder (PTSD) [1].

PTSD is a mental health problem that can occur following the direct experience or witnessing of life-threatening events such as natural disasters, terrorist incidents, serious accidents, or physical or sexual assault in adult or childhood. It is a medically recognized anxiety disorder that occurs in normal individuals under extremely stressful conditions. People who suffer from PTSD often experience symptoms including difficulty sleeping, nightmares and uncontrollable thoughts, feeling estranged from others and depression. The symptoms can be severe enough and last long enough to significantly impair the person’s ability to function in social or family life [2].

The prevalence of PTSD reported in victims of earthquake trauma ranges from 10.3-66.7% [3-5]. Younger age acts as a stronger predictive factor for PTSD, Maercker et al. found that the risk of developing PTSD was greater than the risk of major depression after traumatic events at age 13 or older [6]. As a special group of young adults, college students are at an important period of physical and mental development, their PTSD symptoms and the factors affecting individual susceptibility to PTSD are not well illustrated. This study was conducted to investigate the prevalence of PTSD and correlated risk factors among college students in the senior normal school located in northern Sichuan, the role of psychological interventions in the treatment of PTSD was also evaluated.

## Methods

### Sample

One of the area’s most devastated by the May 12 Wenchuan earthquake was Tibetan autonomous region, a region comprised of 9 towns. At 1 year after the earthquake, 2987 students studying at the senior normal school in this region were selected for this study. To facilitate data collection and management, the students per class were divided into 3 survey groups and each group consisted of 30–50 students, and then an on-site questionnaire investigation was performed.

This study was conducted in accordance with ethical principles stated in the Declaration of Helsinki, 1996 and was approved by the ethical committee of Sichuan University.

### Investigation tools

(1) General information form: demographic data including gender, age, whether injured during the earthquake event, the number of relatives lost in the earthquake, the person’s relationship to victims, and family economic status. (2) PCL-C (PTSD Checklist-Civilian Version), is a standardized self-report rating scale comprising 17 items for assessing PTSD, containing three main kinds of PTSD symptoms: re-experiencing, avoidance, and arousal symptoms. The frequency and intensity of the 17 symptoms of PTSD are rated on a 5 point scale, where 0 indicates that the symptom has not occurred and 4 indicates that the symptom occurs nearly every day. Frequency and intensity scores are multiplied for each of the 17 items and summed to calculate a total score for measuring the severity of PTSD. Scores in the 20–39 are considered in the mild PTSD, 40–59 = moderate PTSD, 60–79 = severe PTSD and ≥ 80 = extreme PTSD [7].

### Investigator training and investigation procedure

The investigators were master degree candidates from West China Medical College of Sichuan University, all of them participated a two-day training course included the study protocol, personal information and collection process, and personal information protection measures. The student filled out their questionnaires under the instruction of the investigators. After being collected, the questionnaires were analyzed by psychiatrists and psychologists.

### Statistics

The sample data were described using the frequency and percentage, the intergroup comparison was performed by the Mann–Whitney *U*-Test, and differences in frequencies and proportions were tested using the chi-square test. Multivariate logistic regression analysis was conducted to examine the associations between PTSD and various socio-demographic variables. Statistical analyses were performed using the SPSS software package (SPSS 16.0, SPSS Inc., Chicago, USA).

## Results

### General data

Among 2987 students, there were 1028 male (34.4%) and 1959 female members (65.6%). The mean (±SD) age was 20.31 (±3.12), with a range from 16 to 26 years old. Twenty-six students (0.9%) were buried in the earthquake ruins, 194 (6.5%) were injured in the earthquake, 18 (0.6%) were hospitalized due to earthquake, and 531 (17.8%) lost their first degree relative; 248 (8.3%) people saw other people being buried in the ruins, 1407 (47.1%) had witnessed other people being wounded and 453 (15.2%) witnessed dead people (Table 1).

**Table 1:** Frequency distribution of study participations

**Characteristic**	**No.**	**%**
Gender		
Male	1028	34.4
Female	1959	65.6
Age (years)		
16 - 20	1479	49.5
21 - 26	1508	50.5
Injured in the earthquake		
Yes	194	6.5
No	2793	93.5
Death of at least one first degree relative		
Yes	531	17.8
No	2456	82.2
Confronting with dead bodies		
Yes	453	15.2
No	2534	84.8
Family income		
Low	2016	67.5
High	971	32.5
Only child		
Yes	1337	44.8
No	1650	55.2
Receiving psychological tutorship		
Yes	447	15.0
No	2540	85.0

The effective questionnaires were subdivided into the severe disaster area and non-severe disaster area according to the locations of students when the earthquake happened, and the severe disaster areas declared by the government included Yingxiu, Beichuan and Qingchuan. Among all the students, 1733 (58.0%) belong to the severe disaster area group. There was no significant difference in the gender ratio between those who lived in the severe disaster and non-severe disaster areas. The mean age of students that lived in the severe disaster area was older than those in the non-severe disaster area, but there was no significant difference (20.7 vs. 19.8, P = 0.089).

### The appearance of PTSD symptoms

The PCL-C scores for the investigated college students were 26.8 ± 9.5, with scores ≥ 20 in 420 cases, and PTSD occurred in 14.1% of the sample. The mean age of 420 PTSD positive students were 20.51 ± 2.26 years. As shown in Table 2, the students were more likely to experience re-experiencing and arousal symptoms one year after the earthquake. More than half of the students had upsetting memories of the earthquake, and these memories could come back when they were not expecting them. Nearly 60% of the students had difficulty concentrating and focusing on a task, and one-third of all college students could not overcome feelings of despair and hopelessness.

**Table 2:** PCL-C quantified form for frequency of different symptoms (N = 2987)

**PCL-C symptoms**	**No.**	**%**
**Re-experiencing symptoms**		
1. Having upsetting memories about the trauma	1722	57.6
2. Experiencing bad dreams and nightmares about the event	966	32.3
3. Feeling as if the trauma were happening again	1580	52.9
4. Getting depressed when reminded of the event	1749	58.6
5. Reacting physically (e.g., sweating, heart racing, trouble breathing) when reminded of the trauma	1030	34.5
**Avoidance symptoms**		
6. Avoiding trauma-related feelings, thoughts, or conversations	1017	34.0
7. Avoiding places, activities, or people that reminded you of the traumatic event	917	30.7
8. Trouble recalling important aspects of what happened during the trauma	769	25.7
9. Losing interest in things you used to enjoy	673	22.5
10. Feeling detached from other people	626	21.0
11. Feeling emotionally numb	406	13.6
12. Feeling as if your future will be cut short	791	26.5
**Arousal symptoms**		
13. Difficulty falling or staying asleep	1245	41.7
14. Experiencing irritability or outbursts of anger	1353	45.3
15. Trouble focusing on tasks	1781	59.6
16. Feeling constantly alert or always on the lookout for danger	1463	49.0
17. Difficulty tolerating and/or easily startled by loud noises	1044	35.0

**Figure 1:**
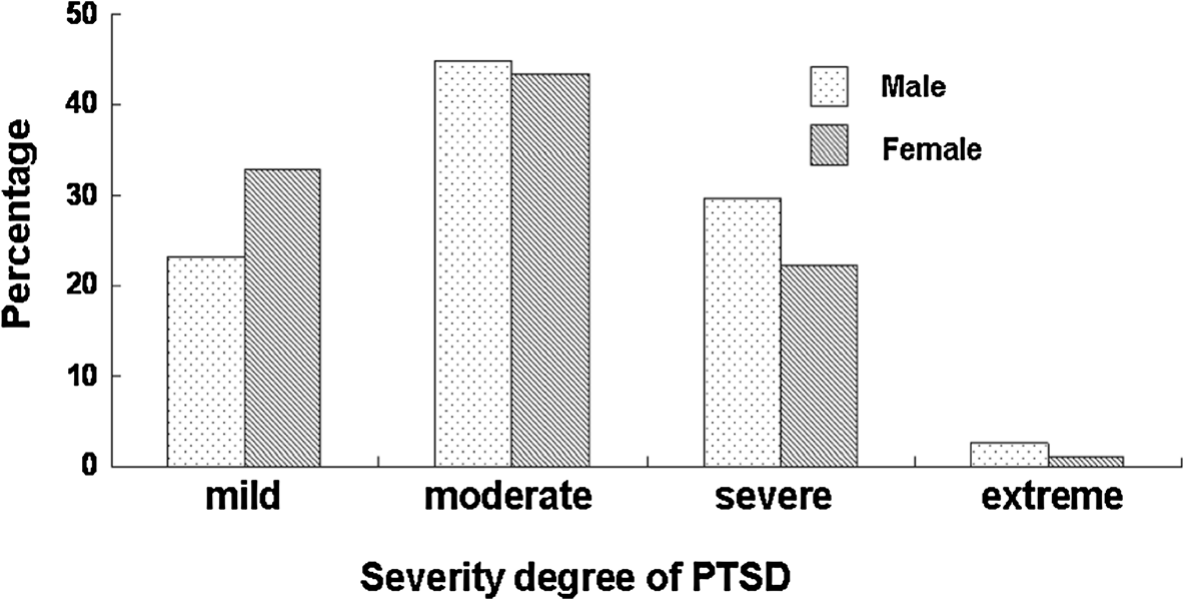
Severity degree of PTSD in male and female college students.

Among all the male students, 165 (16.1%) were diagnosed with PTSD. 38 (23.0%), 74 (44.8%), 49 (29.7%), and 4 (2.4%) respectively, reported mild, moderate, severe and extreme PTSD symptoms. There were a total of 255 (13.0%) female students who were positive for PTSD, among them 84 (32.9%) were graded as mild, 111 (43.5%) as moderate, 57 (22.4%) as severe and 3 (1.2%) as extreme PTSD. More male students were diagnosed as having severe and extreme PTSD, moderate to extreme PTSD symptoms were detected in 77.0% of male students, as compared with 67.1% in female students, and the difference between them was statistically significant (*χ*^2^ = 4.89, P = 0.027) (Figure 1). Among the 420 students diagnosed with PTSD, 309 students lived in severe disaster area and 111 lived in non-severe disaster area, there was a significant difference (*χ*^2^ = 47.79, P < 0.001). The PTSD scores in the severe disaster area group (28.5 ± 9.9) was significantly higher than that in the non-severe disaster area group (24.4 ± 8.4) (t = 11.76, P < 0.001).

### The correlation between PCL-C scores and students’ characteristics

Table 3 showed the final results of the multivariate logistic regression analyses. The students who were injured in the earthquake, those lost their first degree relative, and those confronted with dead bodies were more likely to express PTSD (P < 0.05). In the present study, male students were more prone than female students to develop PTSD (P = 0.046). Students between the ages of 16–20 years old were more likely to express PTSD than those aged ≥ 21 years old, though the difference did not reach statistical significance (P = 0.081). However, the students who received psychological tutorship were less prone to develop PTSD (P < 0.01).

**Table 3:** Multiple regression analysis to assess the correlation between selected characteristics and PTSD occurrence

**Characteristic**	**Estimate**	**SE**	**t**	**P**
Gender	1.031	0.272	−2.086	0.046
Age	−1.617	1.106	3.105	0.081
Injured in the earthquake	2.352	0.971	2.024	0.029
Death of at least one first degree relative	1.016	0.213	2.953	0.001
Confronting with dead bodies	1.920	0.509	3.771	0.000
Family income	4.305	0.817	3.916	0.703
Only child	−3.679	1.529	−4.406	0.192
Receiving psychological tutorship	−1.913	0.462	4.144	0.000

### Social supports and PTSD

After the earthquake, there were 2440 college students who needed economic support and help to meet their basic needs, including food, clothing, and medical care. Among them, 2004 (67.1%) received help from relatives, friends, schools and other social groups. However, 1261 (42.2%) students still felt that the help they got from the society was very little. Only 447 (15.0%) had received psychological tutorship in the investigated college students. Even in the 420 PTSD positive students, only 82 (19.5%) had received psychological tutorship either individually or in small groups.

Positive coping actions were introduced to help to reduce anxiety and lessen distressing symptoms in our study. It is useful for the students to learn about PTSD and how it affects them. By learning how PTSD was, and finding that their problems were shared by millions of mass trauma survivors around the world, they could better recognize that they were not alone and not weak. Practicing relaxation methods such as muscular relaxation exercises, breathing exercises, and meditation were also used, the students who suffered from PTSD could learn to quiet their distress by engaging in relaxing activities. We built a support group, which was comprised of professional counselors and survivors of traumatic experiences, the students with severe or extreme PTSD symptoms were encouraged to talk with them for support, this might help reduce feeling of isolation and rebuild trust in others.

## Discussion

The present study included 2987 college students who experienced direct danger due to a deadliest disaster in modern time, a total of 14.1% of college students met the criteria for PTSD diagnosis one year following the 2008 Wenchuan earthquake. The students who were injured during the traumatic event, those lost their first degree relative, and those confronted with dead bodies had high risks of developing PTSD. Male students were more likely to express PTSD. However, the students who received psychological tutorship were less prone to express PTSD.

The prevalence of PTSD reported in victims of earthquake trauma ranges from 10% to 67%, depending on the nature of the trauma, investigation time and the people that are sampled. In Northridge, California, three months after 6.7 Richter scale earthquake, the incidence of PTSD in the disaster population was 13% [5]. Ten months following the 1999 earthquake in central Taiwan, PTSD was observed in only 10.3% of the population [4]. However, PTSD prevalence rate reached 66.7% in adolescents from the finding of Ziaaddini et al. [3]. Kuo and colleague [8] found that at one year after the Taiwan earthquake, the PTSD rate in survivors was 16.5%, but the investigation on disaster victims with house damage two years after the earthquake had shown that the incidence rate of PTSD reached 20.9%. The above demonstrated that adolescents are more prone than adults to express PTSD.

Age may impact the course of the disorder, children and adolescents are more emotionally vulnerable to the devastating effects of a disaster due to their developmental status [9]. The prevalence of PTSD reached 66.7% in high school students of Bam a city located in Southern Iran, ten months after an earthquake with a magnitude of 6.3 on the Richter scale [3]. We conducted the survey one year after Wenchuan earthquake, and found that 420 developed typical PTSD symptoms in 2987 college students, with a PTSD prevalence rate of 14.1%. The difference amongst previous studies may be due to the different methodologies, sample population and the time of earthquake. The high school students surveyed by Ziaaddini et al. were asleep when earthquake happened. However, we conducted the study among college students who had stronger cognitive skills, the students were attending classes when the event occurred, the teachers could support them emotionally by sharing the horrible experience and encourage them to be strong.

Females were significantly more susceptible to serious psychiatric morbidity than males in some studies conducted on PTSD in post disaster period [10,11], but the differences in the prevalence of PTSD between gender are less clear in adolescents [12]. Our study showed that the incidence rate of PTSD in male was higher than that in the female students. Compared to male students, female students were more willing to acknowledge symptoms and more prone to express their symptoms [13], they would also demonstrate higher average levels of symptoms and seek help more often. A bigger proportional of female students thus received early psychological intervention, resulting in a lower incidence of PTSD.

PTSD prevalence rate was significantly high in severely disastered area, and it was a significant factor associated with PTSD. Preexisting psychopathology and prior exposure to trauma are important individual factors in trauma response [14,15], characteristic of one’s exposure to trauma also significantly influence response. The college students in our study had been living in a safe and peaceful environment, they did not have any practical experience in dealing with big trauma. As victims and on-spot witnesses, the subjects were direct exposures, thus they were more likely to develop severe PTSD.

Poststressor factors significantly influence the severities of PTSD symptomatology [16]. Poststressor factors including the recovery environment, coping methods and treatment provide strong impacts on adjustment following exposure to trauma. Psychological interventions play an important role in the effective treatment of PTSD. We supplied the students who expressed PTSD useful behavioral and cognitive skills to enhance their capacity to manage PTSD-related symptoms. A relatively low rate of PTSD was noted in those who got psychological help following earthquake. Social support is among the strongest predictive factors of PTSD in children and adolescents and may serve as a buffer during and after trauma. For the students who presented with early PTSD symptoms and who have risk factors of PTSD, it is extremely necessary to provide more social support and pertinent psychological help to reduce the occurrence of severe PTSD and prevent chronic PTSD.

## Conclusions

As the most common psychological disorder following a traumatic event, PTSD is a common issue around the world. College students are emotionally vulnerable to the devastating effects of a disaster because of their developmental status. According to the higher proportion of PTSD in the students who were injured and who lost first degree relatives through disaster, more social support and appropriate psychological support should be provided for these high risk groups.

## Competing interests

The authors declare that they have no competing interests.

## Authors’ contributions

JYL and JYH designed the study, YF and CHY coordinated the project. YSC and JW wrote the manuscript. YF and YSC evaluated sample quality control and performed the statistical analyses. XHT, MRJ and GYY participated in the selection process of samples and data collection. All authors read and approved the final manuscript.
